# The health benefits and constraints of exercise therapy for wheelchair users: A clinical commentary

**DOI:** 10.4102/ajod.v6i0.337

**Published:** 2017-09-08

**Authors:** Terry J. Ellapen, Henriëtte V. Hammill, Mariette Swanepoel, Gert L. Strydom

**Affiliations:** 1 School of Biokinetics Recreation and Sport, Physical Activity Sport and Recreation (PhASRec), North-West University, South Africa

## Abstract

**Background:**

There are approximately 1 billion people living with chronic lower limb disability, many of whom are wheelchair users.

**Objectives:**

Review cardiometabolic and neuromuscular risk profiles of wheelchair users, benefits of regular exercise and the causes of neuromuscular upper limb and hip injuries that hinder regular adherence.

**Method:**

Literature published between 2013 and 2017 was adopted according to the standard practices for systematic reviews (PRISMA) through Crossref Metadata and Google Scholar searches. Individual paper quality was evaluated using a modified Downs and Black Appraisal Scale.

**Results:**

The literature search identified 16 600 papers which were excluded if they were non-English, non-peer-reviewed or published before 2013. Finally, 25 papers were accepted, indicating that sedentary wheelchair users have poor cardiometabolic risk profiles (PCMRP) because of a lack of physical activity, limiting their quality of life, characterised by low self-esteem, social isolation and depression. Their predominant mode of physical activity is through upper limb exercises, which not only improves their cardiometabolic risk profiles but also precipitates neuromuscular upper limb overuse injuries. The primary cause of upper limb injuries was attributed to poor wheelchair propulsion related to incorrect chair setup and poor cardiorespiratory fitness.

**Conclusion:**

Wheelchair users have a high body mass index, body fat percentage and serum lipid, cholesterol and blood glucose concentrations. Empirical investigations illustrate exercise improves their PCMRP and cardiorespiratory fitness levels. Although literature encourages regular exercise, none discusses the need to individualise chair setup in order to eliminate wheelchair pathomechanics and upper limb neuromuscular injuries. Wheelchair users must be encouraged to consult a biokineticist or physiotherapist to review their wheelchair setup so as to eliminate possible incorrect manual wheelchair propulsion biomechanics and consequent overuse injuries.

## Introduction

Many wheelchair users suffered injuries to the spinal cord, spinal nerves and cauda equina, and also underwent lower limb amputation (Durstine et al. 2011). According to the International Standards for Neurological Classification for Spinal Injury (Schuld et al. 2014), the anatomical site of the injury determines the categorisation of the spinal cord injury. There are two major categories, namely, tetraplegia and paraplegia. Tetraplegia is identified with neural damage to cervical vertebrae one to seven which produces impairments in both the upper and lower limbs as well as in the trunk, whereas paraplegia is identified with neural damage to the thoracic, lumbar or sacral vertebrae, precipitating trunk and lower limb dysfunction (Schuld et al. 2014).

Lower limb amputation is the surgical or traumatic removal of a person’s lower limbs. The predisposing causes of lower limb amputation include (1) vascular and circulatory diseases precipitated through type 2 diabetes mellitus or peripheral vascular diseases, (2) trauma, (3) surgical removal of tumours and (4) congenital deformities (Durstine et al. 2011). The several classifications of lower limb amputees are (1) symes, (2) transtibial disarticulation, (3) transfemoral disarticulation, (4) hip disarticulation, (5) unilateral amputation and (6) bilateral amputation (Durstine et al. 2011).

The exclusive use of a wheelchair profoundly affects a person’s musculoskeletal and cardiorespiratory functions (Tweedy et al. 2016). These individuals often experience severe depression, which produces social withdrawal and sedentary lifestyles (Jordaan, Swanepoel & Ellapen 2017). Nooijen et al. (2016) reported a high association between a sedentary lifestyle and metabolic syndrome among wheelchair users, increasing their risk of premature death. Although wheelchairs serve as their primary base of support, their source of mobility and as exercise equipment through which they can be physically active, wheelchairs nevertheless limit users’ involvement in physical activity and exercise. The predominant use of the smaller upper limb muscles during manual wheelchair propulsion means that these muscles fatigue easily and also expend less energy in comparison to the larger lower limb muscles (McArdle, Katch & Katch 1996). It is therefore a challenge for wheelchair users to maintain their body fat and body mass index (BMI) levels within normative values (Grogery et al. 2014). Elevated body fat and BMI levels increase their cardiometabolic profile, thereby increasing the onset of obesity, diabetes mellitus, hypertension and of various cardiovascular diseases, as well as of osteoporosis and osteoarthritis (La Fountaine et al. 2015). Grogery et al. (2014) reported an adverse association between prolonged wheelchair sitting and negative cardiometabolic risk profiles. Furthermore, prolonged sitting in wheelchairs has been associated with an anterior pelvic tilt, tight hip flexors and lumbar lordosis, producing lower back pain (Sprigle 2014).

Much empirical research conducted over the last 20 years has reported the effects of sedentary lifestyles of wheelchair users as well as the benefits for those who engage in an active lifestyle (De Groot et al. 2013; Tanhoffer et al. 2014; West et al. 2014). The authors of this paper decided not only to review literature between 2013 and 2017 in order to report on the latest findings but also to include the eight review papers published between 2000 and 2013 (Table 4) so as to reflect the empirical findings of research conducted over the last 20 years (Crtyzer et al. 2013; Da Silva Alves et al. 2013; Grogery et al. 2014; Lu et al. 2014; Nightingale et al. 2017; Oliveira et al. 2014; Sprigle 2014; Tweedy et al. 2016). Previous literature indicates that the physically inactive lifestyle of wheelchair users decreases their basal metabolic rate, and increases their insulin resistance as well as their glucose sensitivity, thereby precipitating the onset of diabetes mellitus together with various other co-morbidities (Grogery et al. 2014; Tweedy et al. 2016; Jordaan et al. 2017). Kressler et al. (2014) and Tanhoffer et al. (2014) have strongly recommended that wheelchair users engage in a physically active lifestyle in order to increase their energy expenditure, thereby decreasing body fat and BMI which will positively influence their cardiometabolic profile. Van Straaten, Cloud and Morrow (2014), Kim et al. (2015) and others have reported that regular exercise and physical activity also diminish muscular and neuropathic pain, thereby improving quality of life.

The objectives of this clinical commentary are to (1) review the cardiometabolic risk profile and cardiorespiratory fitness status of wheelchair users, (2) determine the benefits of regular exercise, (3) determine common neuromuscular injuries adversely influencing wheelchair users adhering to regular exercise therapy and (4) identify wheelchair propulsion pathomechanics as the primary culprit of upper limb overuse and hip injuries. Previous literature encourages wheelchair users to engage in physical activity and exercise but they do not describe the initial challenges (such as muscle and neuromuscular pain and injuries) that users experience. The novelty of this commentary lies in the review of common neuromuscular injuries sustained by wheelchair users when they begin an exercise programme and which may prevent them from continuing with the programme. The identification of the cause of these upper limb overuse injuries among spinal cord injured (SCI) wheelchair users is unique to this review. This is the first commentary to discuss the abnormal force-couple relationships of the shoulder and hip muscles because of poor wheelchair setup and propulsion pathomechanics.

## Methodology

The authors followed the standard practices for systematic reviews (PRISMA). The definitions were guided by the PRISMA checklist for participants, interventions, comparisons, outcomes and study designs (PICOS). The participants in this study were wheelchair users; the intervention was not necessarily a therapeutic intervention but is interpreted as an exposure, namely, the effect of exercise therapy on the well-being of wheelchair users. The outcomes of interest were (1) exercise therapy interventions for wheelchair users, (2) the effects of exercise therapy on wheelchair users’ health and (3) common overuse injuries of physically active wheelchair users. The exclusion criteria were (1) publications prior to 2013, (2) literature not related to the health and physical status of wheelchair users, (3) psychological therapeutic interventions, (4) non-English papers and (5) non-peer-reviewed papers.

**FIGURE 1:**
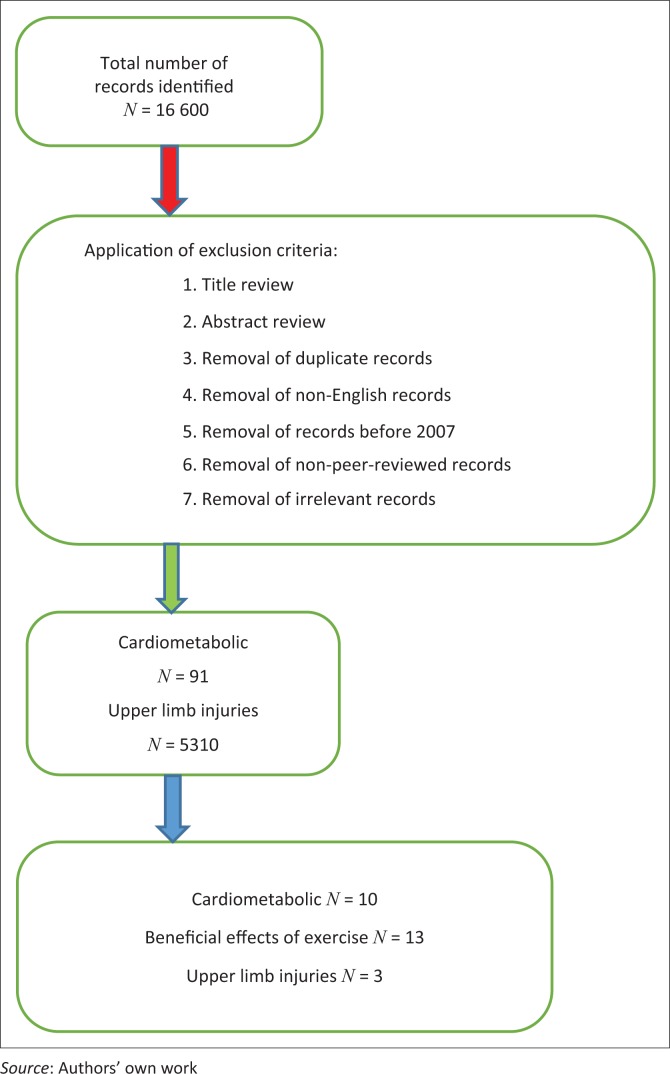
Conceptualisation of the review process.

A literature search of peer-reviewed and professional journal publications was conducted in the following search engine: Crossref Metadata database, an academic meta-database which comprises the following search engines: PubMed, Medline, Science Direct, Ebscohost, CINAHL and Google Scholar (Figure 1). The keywords used in the literature search were wheelchair users, physiological limitations of wheelchair users, impact of exercise therapy on wheelchair users’ health and quality of life. The screening eligibility of papers was performed in the following three steps: (1) title screen, (2) abstract screen and (3) full-text screen. The papers were screened by T.J.E., H.VH. and M.S.

## Hierarchy of evidence and quality appraisal

The hierarchy of evidence and quality of appraisal tool were adapted from Abdullah, McDonald and Jaberzadeh (2012) (Table 1). All publications were filtered based on the appropriateness of their title and whether they met the inclusion criteria. The authors included all levels of evidence as long as the publications met the inclusion criteria because of the limited literature available.

**TABLE 1:** Hierarchy of evidence.

Levels	Types of literature
Level 1	Systematic review
Level II-1	Randomised control trials
Level III-1	Pseudo-randomised controlled trial
Level III-2	Comparative study with concurrent controls
Level III-3	Comparative study without concurrent controls
Level IV	Case series/studies with either post-test or pre-test/post-test outcomes

*Source*: Abdullah et al. 2012

The quality of each paper was appraised using a modified Downs and Black Appraisal Scale, which examined the quality of randomised controlled trials and non-randomised papers (Downs & Black 1998) (Table 2). The modified Downs and Black checklist was adopted as not all the items on the original checklist were related to this paper, as similarly cited in Gorber et al. (2007) and in Ellapen, Paul, Swanepoel and Strydom (2017). The modified checklist comprised 15 questions with a maximum of 15 points. Answers were given a score of either 0 or 1. The authors did not adopt the PEDro Scale because it primarily focuses on the appraisal of randomised control trials (PEDro Scale 1999).

**TABLE 2:** Appraisal of the papers selected according to Downs and Black Appraisal Scale (*n* = 25).

Authors	Reporting (*n* = 8)	External validity (*n* = 3)	Internal validity (*n* = 3)	Power (*n* = 1)	Total (*n* = 15)
Crtyzer et al. (2013)	4	1	1	1	7/15
Da Silva Alves et al. (2013)	4	0	1	1	6/15
De Groot et al. (2013)	4	2	2	1	9/15
Froehlich-Grobe et al. (2014)	5	2	3	1	11/15
Grogery et al. (2014)	3	2	1	1	7/15
Kressler et al. (2014)	6	2	3	1	12/15
Lu et al. (2014)	4	1	1	1	7/15
Oliveira et al. (2014)	4	1	1	1	7/15
Sprigle (2014)	4	0	1	1	6/15
Tanhoffer et al. (2014)	4	2	2	1	9/15
Van Straaten et al. (2014)	5	1	2	1	9/15
West et al. (2014)	5	2	3	1	11/15
Kim et al. (2015)	5	2	3	1	11/15
La Fountaine et al. (2015)	5	2	2	1	10/15
Thompson, Snodgrass and Osmotherly (2015)	4	2	2	1	9/15
Van Der Scheer et al. (2015)	5	2	2	1	10/15
Vosloo, Ntsiea and Becker (2015)	6	2	2	1	11/15
Will et al. (2015)	4	2	3	1	10/15
Wong et al. (2015)	5	1	2	1	9/15
Chen et al. (2016)	5	2	3	1	11/15
Nooijen et al. (2016)	5	2	3	1	11/15
Torhaug et al. (2016)	5	2	3	1	11/15
Tweedy et al. (2016)	3	1	1	1	6/15
Nightingale et al. (2017)	4	1	1	1	7/15
Ekelman et al. (2017)	4	2	1	0	7/15

*Source*: Authors’ own work

## Results

The database searches identified 16 600 full-text articles. After stringent application of the exclusion criteria, 25 eligible papers were incorporated into this clinical commentary. The findings of the chronological review of literature, identifying the cardiometabolic risk profiles and benefits of exercise for wheelchair users with SCI, and the review papers published between 2013 and 2017 are summarised in Tables 3 and 4, respectively.

**TABLE 3:** Chronological review of literature, identifying the cardiometabolic risk profiles and benefits of exercise for wheelchair users with spinal cord injuries (2013–2017) (*n* = 17).

Authors	Sample characteristics	Objective of study	Findings
De Groot et al. (2013)	*N* = 130 Age: 40.1 ± 13.8 years	To investigate the prevalence of coronary heart disease risk factors among individuals with SCI.	Sedentary lifestyle is one of the contributing factors for cardiometabolic risk for individuals with SCI.
Froehlich-Grobe et al. (2014)	*N* = 118 (RCT) Age: 44.5 ± 1.1 years	To compare the adherence of wheelchair users with SCI to two home exercise programmes over 12 months.	Individuals with SCI who received psychological support with exercise therapy had a higher level of adherence than those who did not have this support.
Kressler et al. (2014)	*N* = 5 circuit training *N* = 6 circuit training and protein supplementation Age: not specified	To determine the effectiveness of circuit training programmes using selected fitness parameters among wheelchair users with SCI.	Regular circuit training improves VO_2peak_, power output and muscle strength of individuals with SCI.
Tanhoffer et al. (2014)	Exercise group = 6, Sedentary group = 7 Age: 40 ± 13 years	To determine the long-term effect of exercise on the energy expenditure of individuals with SCI.	Individuals with SCI who exercise regularly have a higher energy expenditure and lower body fat level than sedentary counterparts.
Van Straaten et al. (2014)	*N* = 16 Age: 41 years	To determine the effect of a shoulder strengthening programme regarding the neuropathic pain of wheelchair users with SCI.	Shoulder strengthening exercises reduced neuropathic pain.
West et al. (2014)	*N* = 10 wheelchair rugby players Age: 30.5 ± 2.2 years	To determine the effectiveness of specific inspiratory training on respiratory structure, function and peak exercise response in wheelchair users with SCI.	Inspiratory training increases respiratory muscle size and strength, which enhances the aerobic performance of individuals with SCI.
Kim et al. (2015)	Experimental group = 8 Control group = 7 Age: not specified	To investigate the effects of a six-week indoor hand-bike exercise programme on fasting insulin levels and physical fitness levels in wheelchair users with SCI.	Exercise using an indoor hand-bike improves body composition, fasting insulin and fitness levels among individuals with SCI.
La Fountaine et al. (2015)	*N* = 145 Age: 41.3 ± 11.6 years	To determine the effect of prolonged sitting and limited physical activity on cholesterol status for wheelchair users with SCI.	Prolonged sitting and limited physical activity negatively affect cholesterol status of individuals with SCI.
Thompson et al. (2015)	*N* = 278	To determine the prevalence of injuries in wheelchair sport in comparison to the benefits.	Although wheelchair sport does produce injuries, specifically to the upper limb, there are many benefits.
Van der Scheer et al. (2015)	*N* = 40 Age: 18–65 years	To determine the effects of low-intensity wheelchair strengthening exercises on the fitness and physical activity levels of sedentary wheelchair users with SCI.	Low-intensity aerobic wheelchair exercises improve the cardiorespiratory function of individuals with SCI.
Will et al. (2015)	*N* = 29 experimental *N* = 14 control	To investigate the effects of low-intensity propulsion training on wheelchair biomechanics.	Low-intensity wheelchair propulsion training does not improve wheelchair biomechanics.
Wong et al. (2015)	*N* = 645 Age: not specified	To examine the attitudes, knowledge and perception of medical staff concerning individuals with SCI.	Individuals with SCI need to engage in an obesity prevention intervention programme in order to improve their cardiometabolic risk profile and quality of life.
Vosloo et al. (2016)	*N* = 45 (individuals with SCI) *N* = 21 (able-bodied)	To determine ambulation energy expenditure and factoring AEE among individuals with SCI compared to able-bodied individuals.	Walking energy expenditure of able-bodied people is higher than that of individuals with SCI. Hip flexion contracture limited the ability of individuals with SCI to walk, which reduced their caloric expenditure.
Chen et al. (2016)	*N* = 138	To test the effects of a six-month elastic band exercise programme on daily living and functional fitness of elderly wheelchair users.	Elastic band exercises improved the activities of daily living and functional fitness of wheelchair users.
Nooijen et al. (2016)	*N* = 45 Age: 44 ± 15 years	To determine whether rehabilitation reinforced with a behavioural intervention promotes physical activity, thus leading to a more active lifestyle than rehabilitation alone for individuals with SCI.	Individuals with SCI need psychological motivation in order to adhere to regular physical activity.
Torhaug et al. (2016)	*N* = 11 experimental *N* = 6 control	To assess the effect of maximal bench press strength training on wheelchair propulsion work economy.	A six-week maximal bench press strengthening programme significantly improved wheelchair economy during wheelchair propulsion.
Ekelman et al. (2017)	*N* = 4 Age: 39 ± 9.9 years	To determine the impact of a wellness programme on the physical, social and mental well-being of individuals with SCI.	Participation in a wellness programme positively influenced the physical, mental and social well-being of the participants.

*Source*: Authors’ own work

SCI, spinal cord injuries; AEE, ambulation energy expenditure.

**TABLE 4:** Review papers of wheelchair users with spinal cord injuries (2013–2017) (*n* = 8).

Authors	Sample characteristics	Findings
Crtyzer et al. (2013)	Review	Individuals with spina bifida have a sedentary lifestyle, lower aerobic capacity and higher prevalence of obesity. Therapeutic exercise interventions reduced pain, increased biomechanical efficiency during wheelchair propulsion and increased physical activity levels and improved balance.
Da Silva Alves et al. (2013)	Review	Exercise reduces SCI inflammation.
Grogery et al. (2014)	Review	Physical inactivity among individuals with SCI adversely impacts their metabolic profile, leading to the development of co-morbidities such as diabetes mellitus and cardiovascular diseases.
Lu et al. (2014)	Review	Successful treatment of individuals with SCI should include exercise therapy and functional electrical stimulation of the upper limb in an attempt to improve muscle strength, cardiometabolic risk profile, upper limb function, as well as participation in activities of daily living and their quality of life.
Oliveira et al. (2014)	Review	A limited amount of literature pertaining to spina bifida and exercise exists. However, the literature does indicate that exercise enhances the cardiorespiratory function and muscle strength of individuals with SCI.
Sprigle (2014)	Review	Identified the need for proper wheelchair fit, as well as the ramifications of incorrect wheelchair fit upon the patient.
Tweedy et al. (2016)	Review	Exercise interventions improve the cardiorespiratory fitness, and decrease the cardiometabolic risk profile, pain and depression levels of individuals with SCI.
Nightingale et al. (2017)	Review	Exercise is mainly limited to the upper body, which involves a smaller activated muscle mass. Current exercise guidelines for SCI focus predominantly on relatively short durations of moderate intensity aerobic arm cranking exercise; yet evidence indicates that this is inadequate for the reduction of cardiometabolic disease risk factors. It is proposed that high-intensity interval training may be a viable alternative exercise strategy in order to promote vigorous intensity exercise and prevent cardiometabolic disease in persons with SCI.

*Source*: Authors’ own work

SCI, spinal cord injuries.

## Discussion

The discussion will follow the order of (1) cardiometabolic risk profile, (2) benefits of regular exercise to wheelchair users and (3) common neuromuscular injuries from upper extremity exercises.

### Cardiometabolic risk profile of wheelchair users

Wheelchair users often lead sedentary lifestyles and consequently have poor cardiometabolic profiles (high BMI, increased body fat percentage and abnormal lipid and glucose concentrations) (Grogery et al. 2014; La Fountaine et al. 2015; Nooijen et al. 2016). Many wheelchair users are classified as obese. Normal to excessive eating coupled with the minimal levels or absence of regular physical activity of wheelchair users, increases their body fat and BMI, both of which are predictors of obesity (Grogery et al. 2014). McArdle et al. (1996) and Jordaan et al. (2017) reported that the sedentary lifestyle of wheelchair users decreases their basal metabolic rate and glucose sensitivity, as well as increases their insulin resistance, thereby precipitating the onset of diabetes mellitus and ultimately contributing to their poor cardiometabolic profile. Their poor cardiometabolic profile increases the risk of various cardiovascular diseases and metabolic syndrome (Durstine et al. 2011). Furthermore, their cardiorespiratory capacity is markedly reduced in so far as their condition is often accompanied by atrophied or weak respiratory muscles (West et al. 2014). The lack of regular physical activity and exercise, which is one of the hallmarks of sedentary individuals, is attributed to reduced maximal oxygen consumption, thereby limiting one’s aerobic capacity. Because of the relationship between the cardiovascular and respiratory systems, a reduced aerobic capacity adversely influences a person’s cardiorespiratory capacity (McArdle et al. 1996). Wheelchair users might furthermore be depressed, experiencing low-esteem and becoming socially withdrawn (Nightingale et al. 2017), which negatively impacts their willingness to exercise and probably contributes to an increased cardiometabolic risk profile.

### Benefits of regular exercise

The World Health Organization (WHO 2016) recommends regular exercise participation of low to moderate intensity, either of physical or recreational activities, at least three times per week for approximately 30 min a day. The exercise can be aerobic or resistance training or a combination of both. Benefits from participation in regular exercise include:

- Regular aerobic training, applying manual wheelchair propulsion, arm cranking, swimming and circuit training have proven to increase the cardiorespiratory fitness, upper extremity muscle strength and endurance of wheelchair users (Kressler et al. 2014; Torhaug et al. 2016; Tweedy et al. 2016). Wheelchair users who regularly exercise have higher cardiorespiratory fitness, better cardiometabolic profiles (decreased BMI, percentage of fat and lipids) and tend to frequently participate in daily activities such as personal grooming, cleaning their surrounding environments and wheelchair riding (Tanhoffer et al. 2014). Aerobic exercise increases maximal oxygen consumption, thereby improving cardiorespiratory status through the efficient transportation of oxygen and carbon dioxide through the cardiovascular system both to and from the exercising muscles. Further aerobic exercises help to decrease high blood glucose, body fat and BMI levels, which improves a person’s cardiometabolic profile (McArdle et al. 1996; Van der Scheer et al. 2015).
- Kressler et al. (2014) and Zolot and Rosenberg (2016) reported that regular circuit training improves VO_2peak_, power output and muscle strength. Circuit training utilises the short-term energy system that predominantly stimulates fast oxidative glycolytic fibres, increasing muscle strength and endurance (McArdle et al. 1996). Increased muscle strength and endurance improve wheelchair user’s daily living activities and quality of life (Tanhoffer et al. 2014).
- West et al. (2014) reported that regular inspiratory and aerobic exercise elicits improvements in respiratory functioning. This enhanced cardiorespiratory adaptation can be useful in order to prolong upper extremity aerobic training, which in turn will increase caloric energy expenditure and reduce their body fat percentage.
- Regular exercise reduces depression and improves quality of life among these individuals (Tweedy et al. 2016; Zolot & Rosenberg 2016).
- Da Silva Alves et al. (2013) and Van Straaten et al. (2014) reported that regular exercise reduces spinal cord injury inflammation and neuropathic pain.

### Common neuromuscular injuries affecting regular exercising of spinal cord injured individuals

The WHO reported that 15.6% of the world’s population (approximately 1 billion people) are living with chronic disability and spend a considerable amount of time in wheelchairs (Kate 2015; WHO 2016). Most of these people are sedentary but a small portion of wheelchair users forgo a sedentary lifestyle and are instead physically active, using their wheelchairs as exercise apparatus. Certain challenges, such as upper limb overuse injuries, inhibit these individuals from pursuing physical activity through wheelchair mobility. These upper limb overuse injuries pose further limitations on their already restricted lifestyle (Thompson et al. 2015). The following discussion will entail what the pathomechanics are of common overuse upper extremity injuries among wheelchair users.

#### Pathomechanics of common overuse upper extremity injuries among wheelchair users

The most common overuse injuries include shoulder impingement, rotator cuff tendinitis, biceps tendinitis, lateral epicondylitis, ulnar neuropathy, De Quervain’s tenosynovitis and carpal tunnel syndrome (Apple, Cody & Allen 2004). Will et al. (2015) reported that poor biomechanics adopted in propelling wheelchairs is the primary cause of these overuse injuries. Van der Scheer et al. (2015) reported that poor fitness conditioning status among wheelchair users precipitates poor wheelchair propulsion biomechanics, which in turn leads to upper limb overuse injuries.

Manual wheelchair propulsion is categorised by the contact and recovery phases. Contact phase occurs when mechanical power is delivered to the wheelchair through hand contact with the rim of the wheel (Slowik et al. 2016). During the recovery phase, the hand is repositioned in preparation for the next cycle. During the subsequent contact phase, the hand is constrained to the arc of the rim of the wheel. Contact phase hand patterns include distinct hand pattern types, which are based on the shape of the projection onto the rim arc and can be grouped into the following patterns: single loop, double loop and semi-circular loop (Slowik et al. 2016). Qi et al. (2014) reported that the selection of hand pattern influences the onset of upper extremity pain and injury.

**FIGURE 2:**
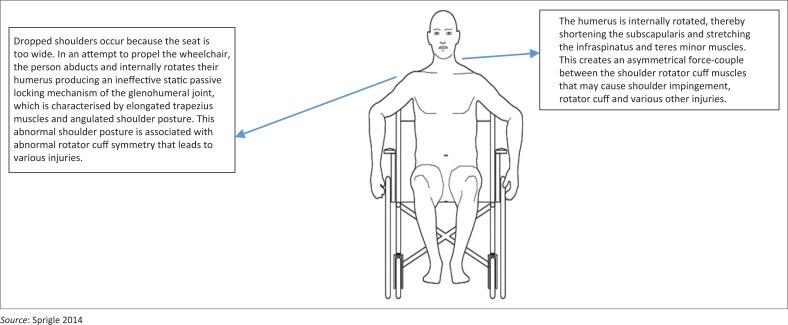
Frontal plane analysis identifying dropped shoulders.

Sprigle (2014) and Will et al. (2015) stated that the key aspects of poor biomechanical posture among wheelchair users who manually propel their wheelchairs are: forward leaning and dropped or drooping shoulders. An anterior frontal plane analysis of the aforementioned pathomechanics indicates that a dropped or angulated shoulder girdle posture is associated with various shoulder pathologies (Figure 2). From this image of poor shoulder girdle posture, it is postulated that these individuals are experiencing the phenomenon known as the ‘ineffective static passive locking mechanism of the glenohumeral joint’. Mansfield and Neumann (2015) describe the ineffective static locking mechanism as occurring because of scapular depression and downward rotation because of the laxed superior glenohumeral capsule and eccentrically lengthened trapezius and rhomboid muscles. This creates an abnormal force-couple relationship between the lengthened trapezius and the shortened pectoralis minor in the frontal plane (Mansfield & Neumann 2015). The shortened pectoralis minor muscles also produce a sunken chest and kyphosis. In the sagittal plane, the rounded shoulder suggests pectoralis minor and serratus anterior contractures as well as lengthened rhomboids. Furthermore, the humeral head is depressed and internally rotated indicating subscapularis contracture (Mansfield & Neumann 2015). The ineffective static locking mechanism decreases the impingement interval space between the humeral head and the coracoamical arch and this reduced impingement interval spacing produces compression of the sub-acrominal bursa, supraspinatus and biceps brachii (Mansfield & Neumann 2015). The aforementioned biomechanical cascade of events explains the pathomechanics of shoulder impingement, sub-acrominal bursitis, rotator cuff injury (supraspinatus tear) and biceps tendinopathy (Mansfield & Neumann 2015). It is recommended that symmetrical strengthening of the trapezius, rhomboid, teres minor and infraspinatus muscles and stretching of the subscapularis, pectoralis minor and serratus anterior be undertaken in order to restore correct shoulder girdle posture, increasing the impingement interval space, thereby alleviating soft tissue sub-coracoamical arch compression. Sprigle (2014) recommended that the seat width, seat depth, seat height, footrest length, armrest height, backrest height and backrest upholstery of the wheelchair be ergonomically adjusted so as to meet the individual’s needs and prevent poor biomechanical posture and overuse injuries.

**FIGURE 3:**
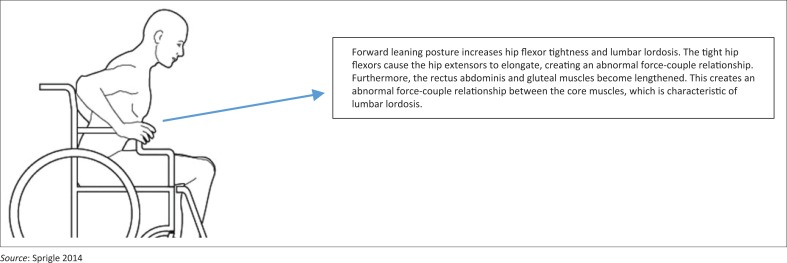
Sagittal plane analysis identifying poor posture.

Sagittal plane analysis identifies an anterior pelvic tilt with increased hip flexor tightness and lumbar lordosis (Sprigle 2014) (Figure 3). An anterior tilted pelvis is also known as short-arc pelvis-on-femur flexion and is associated with short tight hip flexors (iliopsoas and rectus femoris) and elongated stretched gluteal muscles (Mansfield & Neumann 2015). This creates an abnormal force-couple asymmetrical relationship between the hip flexors and extensors that leads to lower back pain (Mansfield & Neumann 2015). Furthermore, tight hip flexors are associated with lumbar lordosis which is characterised by hyperextension of the lumbar vertebrae, accompanied by tight short erector spinae and stretched elongated rectus abdominis muscles and lower back pain (Mansfield & Neumann 2015).

## Evolution of wheelchair design

In an attempt to reduce wheelchair propulsion pathomechanics, scientists and engineers have redesigned the appearance and functionality of wheelchairs. Sports wheelchairs have undergone drastic and revolutionary design modifications in order to enhance sports performance and improve adherence to physical activity programmes. These ergonomic modifications improve the biomechanics of the user, which in turn curtails the incidence of upper limb overuse injuries (Sindall et al. 2013). Modern tennis wheelchairs have sharply slanted back wheels so that the player is able to change direction easily (Sindall et al. 2013). The seat height of the wheelchairs for basketball-forwards has been raised, while the guards’ wheelchairs have an inclined seat so as to facilitate improved wheelchair propulsion biomechanics (Sindall et al. 2013). Although sports wheelchairs are used for relatively short or temporary durations in the course of a wheelchair user’s day, it is nevertheless important to acknowledge the progress that has been made in wheelchair design and functionality. These revolutionary wheelchair design modifications have been embraced by scientists and engineers who are redesigning the normal manual wheelchairs that are utilised for a longer duration by incorporating similar features to those which have been brought to sports wheelchairs (Cloud et al. 2017). Cloud et al. (2017) have redesigned the seat angle of normal manual wheelchairs and have thereby significantly reduced the anterior pelvic tilt and lumbar lordosis of potential users. Smart and powered wheelchairs provide on-board navigation and electronic transmission in order to enable the user to adopt an independent lifestyle without much physical effort (Leaman & Hung 2015). The authors postulate that because of the limited need for physical activity in these wheelchairs, the sedentary nature of the user’s lifestyle will be further increased, which in turn may adversely affect their cardiometabolic profile.

## Recommendations

It is recommended that manual wheelchair users have their wheelchairs reviewed in order to ensure that the wheelchair setup is ergonomically designed to meet their individual needs. Treatment and rehabilitation of the aforementioned overuse injuries pose a significant challenge because these individuals are primarily reliant on the upper limbs for weight-bearing activities and for mobility. It is further recommended that before starting an exercise programme, all wheelchair users should first receive clinical clearance from their medical practitioner regarding their participatory readiness. They must thereafter consult a biokineticist or a physiotherapist who will conduct a critical review of their wheelchair propulsion biomechanics in an attempt to prevent injuries. The biokineticist or physiotherapist should also prescribe an individualised therapeutic exercise programme.

## Conclusion

Wheelchair users have poor cardiometabolic risk profiles, low self-esteem and are at risk for socially withdrawn lives. Those who regularly exercise enjoy improved cardiorespiratory fitness and reduced cardiometabolic risk as well as reduced levels of depression and a consequently enhanced quality of life. Unfortunately, many wheelchair users who wish to be physically active are further restricted by upper limb overuse injuries. The primary cause of these injuries is wheelchair propulsion pathomechanics as a result of incorrect chair setup and limited cardiorespiratory fitness. It is therefore recommended that wheelchair users consult a biokineticist or physiotherapist before engaging in an exercise regime, so as to alleviate poor wheelchair propulsion biomechanics which may predispose them to overuse injuries. Medical practitioners, as well as the family and friends of wheelchair users, must encourage them to adhere to regular aerobic, muscle strength, and flexibility exercises in order to improve their quality of life.
